# Setting up a pharmacy HIV pre-exposure prophylaxis delivery model: Lessons and recommendations for implementation

**DOI:** 10.4102/sajhivmed.v26i1.1683

**Published:** 2025-03-25

**Authors:** Tsitsi Nyamuzihwa, Kelechi E. Oladimeji, Athini Nyatela, Lettie Makola, Samanta T. Lalla-Edward, Angela Tembo

**Affiliations:** 1 Ezintsha, Faculty of Health Sciences, University of the Witwatersrand, Johannesburg, South Africa

## Introduction

Since its registration in South Africa in 2016, the uptake of oral pre-exposure prophylaxis (PrEP) for HIV prevention has been slow, and PrEP continuation remains a challenge.^1,2^ PrEP is primarily delivered through public healthcare facilities and community settings. Barriers to PrEP uptake include long travel distances, extended waiting times, and stigma.^3^ To overcome these challenges, the WHO recommends differentiated service delivery models, which tailor services to individual preferences to expand PrEP access.^4^

Community pharmacies represent an underutilised potential service delivery model for expanding PrEP access, both in South Africa and globally.^5^ Research on PrEP delivery in community pharmacies in South Africa is limited. However, implementation studies conducted in Kenya and the United States have found that community pharmacy-based PrEP delivery models are feasible, acceptable, and reach at-risk individuals.^5,6^ South Africa has a well-established community pharmacy footprint with approximately 3580 registered pharmacies in easily accessible locations, offering extended hours and a less stigmatised setting, thus making them ideal for PrEP delivery.^7^

Our implementation project aims to assess the acceptability and feasibility of pharmacy delivered PrEP.^8,9^ The project is donor funded and underway in 10 community pharmacies. This article discusses our preliminary learnings drawn from various activities including formative work, stakeholder consultations, and market research in the period September 2022 and March 2023. The article highlights critical issues to consider for future pharmacy-delivered PrEP models with a summary of the narrative provided in Online Appendix 1.

## Designing the community pharmacy pre-exposure prophylaxis model

Two main issues required attention: (1) legislative barriers to implementation, and (2) establishing efficient patient management systems within the pharmacy setting:

- The South African Pharmacy Council approved a Pharmacist Initiated Management of Antiretroviral Therapy (PIMART) course for pharmacy personnel, but legislation for prescribing permits is under legal review.^10^ To overcome this barrier, the project utilises a telemedicine platform to support PrEP scripting. However, PIMART remains important because it provides adequate training and autonomy for pharmacy staff to prescribe PrEP once the legal process is resolved.
- A round table meeting with key stakeholders from different sectors was convened on 22 November 2022, with the main objective of addressing potential operational pitfalls. A total of 28 people attended: nine from research institutions, six from funding agencies, six from the National Department of Health and public health agencies, four from community pharmacy groups, and three from the private sector. Community pharmacy representatives proposed the use of in-house data systems for patient management and communication. All attendees emphasised the importance of providing comprehensive sexual reproductive health services with PrEP. Digital support mechanisms for follow-up and a pharmacy self-care package for HIV screening were supported.

Following the meeting, the project team participated in a behavioural design workshop aimed at improving uptake and persistence on oral PrEP among gay men and other men who have sex with men.^11^ Insights from the workshop assisted in the development of a simplified pharmacy–client user journey incorporated into pharmacy workflows. Recommendations included utilising digital tools to expedite procedures, courier delivery of PrEP, and reviewing clinical investigations required at initiation per national PrEP guidelines.

## Recruiting pharmacies

A geographic information system mapping process was used to identify pharmacies located in communities that would benefit from PrEP. In this multi-step process, service delivery gaps were identified and the total number of people remaining on antiretroviral therapy (ART) in public facilities (used as an indication of HIV burden) overlaid with the nearest pharmacies in the Gauteng and Western Cape subdistrict regions. In the final step, a map of these pharmacies was overlaid with high foot traffic areas such as shopping centres, transport hubs, and institutions of higher learning: all considered ideal for easy access to PrEP services and proximity to at-risk populations. The list of pharmacies was narrowed in consultation with pharmacy groups with preference for pharmacies that had PIMART-trained pharmacy personnel, where possible. Of the final 10 community pharmacies selected, six are in Cape Town, two in Johannesburg, and two in Tshwane. Six are independently owned and four are corporate pharmacies. Of the total, four are in central business districts, three are close to institutions of higher learning, two in shopping malls, and one in a township.

Engagements with the pharmacy groups highlighted several important factors for project buy-in. Aligning business and marketing strategies for PrEP services with key decision- makers within the organisation was crucial for corporate groups, while independent owners required a well-defined reimbursement structure for commodities and services to ensure participation.

The recognition of pharmacy providers as healthcare providers reimbursed for time spent in bettering health outcomes have also been highlighted as a requirement for expanding pharmacists’ scope in HIV prevention in the American community pharmacy setting.^12^ Finally, integrating the reimbursement of participant PrEP costs (drugs and commodities) from project funding in existing pharmacy systems was important for operational efficiency.

## Clinical monitoring systems

Access to laboratory facilities for clinical investigations, specifically hepatitis B and creatinine where indicated, is required for PrEP initiation.^13^ In addition to dispensing medications, most community pharmacies provide primary health clinic services such as immunisations, contraception, and health screenings. Because of this limited scope of clinical services and the absence of trained staff for phlebotomy, laboratory facilities and/or logistics systems for transportation of samples are not available in most community pharmacies. A laboratory service provider supported by a courier system was contracted, although this increased costs, particularly for pharmacies in locations outside the collection radius of the provider and thus charged an extra fee for courier of samples. While utilising pre-existing structures is ideal, these adjustments may be necessary to deliver PrEP services. Recommendations include training staff on sample collection and service level agreements with laboratory providers. Pharmacy owners might also consider investing in point-of-care devices for long-term convenience and reduced logistics costs.

## Situational assessments: infrastructure and resources

The availability of infrastructure and resources are important facilitators of community pharmacy PrEP delivery.^14^ An audit of infrastructure and resources using a readiness tool developed for the project looked at availability of pharmacy clinic rooms, presence of patient record systems, and clinically trained staff. Most pharmacies had adequate space and private consulting rooms, but some required more human resources. Nurses were hired to assist with PrEP initiation in pharmacies that needed this resource, highlighting the need to ensure sufficient capacity for the provision of services. All pharmacies had electronic data management systems and follow-up strategies for medication refills. Utilising these existing systems for PrEP provision facilitates seamless integration and enhances healthcare worker acceptance of the model.

## Pre-exposure prophylaxis knowledge and skills

Formative work, which included in-depth interviews, was conducted with 11 healthcare providers at baseline. Providers were purposively selected to include at least one pharmacist and/or nurse from pharmacy sites in Johannesburg and Cape Town. The open-ended question guide aimed to ascertain prior training and experience with PrEP initiation. One key theme that emerged from the thematic analysis of interviews with providers was the identification of knowledge and skills gaps related to administering PrEP, specifically, the providers’ understanding of PrEP protocols, counselling skills, competence in managing side effects and PIMART training exposure. Of the 11 providers interviewed, over 70% reported that they were not PIMART trained. In response to this, the project team enrolled staff in PIMART training. A total of 34 pharmacy staff were enrolled for training, and 14 completed training before the start of the project. Ongoing evaluations show that PIMART training improved staff competency and boosted their confidence in initiating clients on PrEP. The long-term benefits of training programmes such as PIMART include improved HIV service delivery and increased uptake of PrEP.^14^ These findings underscore the importance of training and development for effective pharmacy-led PrEP programmes.

## Demand creation

Findings from the formative interviews indicated poor PrEP knowledge among pharmacy clients. Limited awareness and low perceived risk of HIV infection pose challenges to reaching populations who could benefit from PrEP.^15^ As a result, there was great emphasis on demand creation activities during the planning phase of the project. Pharmacy owners and staff were engaged to create tailored demand creation strategies, using combinations of in-house advertising, social media, printed materials, campus and community outreach, and person-centred approaches (e.g. offering PrEP to clients accessing sexual and reproductive health services). Continuous assessment of client and community needs is essential for effective demand creation.

## Conclusion

Lessons learned from setting up this novel PrEP delivery model indicate that community pharmacies in South Africa have the adequate infrastructure, patient management systems and proximity to at-risk population groups that can benefit from PrEP. Despite operational challenges such as setting up logistics systems for laboratory investigations and challenges with PIMART legislation, community pharmacies participating in the project are adequately equipped to deliver PrEP to the public. The presence of trained personnel remains critical in ensuring delivery of quality PrEP services and continued advocacy for PIMART legislation remains a priority. Strategies for scale-up post donor funding should include public–private partnerships to subsidise the costs of PrEP to clients such as the use of state drugs and consumables.
